# Study on the detonation wave propagation of shaped charge with three-layer liner and its driving characteristics to liner

**DOI:** 10.1038/s41598-024-59402-y

**Published:** 2024-04-16

**Authors:** Zhiwei Hao, Zhijun Wang, Yongjie Xu, Conghui Duan, Yifan Wang

**Affiliations:** 1 College of Mechanical and Electrical Engineering, North University of China, Taiyuan, 030051 China; 2 Chongqing Hongyu Precision Industrial Co.,Ltd., P.O.Box 402760, Chongqing, People’s Republic of China

**Keywords:** Three-layer liner, Jet, Impact impedance, Detonation wave, Orthogonal test, Penetration depth, Numerical simulation, Engineering, Materials science

## Abstract

With the continuous improvement of various armor protection technologies, the armor protection performance has increased significantly, and then the damage performance requirements of armor-piercing ammunition have also increased. In order to improve the penetration ability of the liner, a new three-layer liner structure is designed in this paper. The jet forming process was simulated by AUTODYN software. The mechanism of shaped jet forming of three-layer liner was studied. The reason why the penetration depth of three-layer liner was higher than that of ordinary liner was explained. The influence of three-layer liner on the propagation of detonation wave and the change of pressure when detonation wave acted on liner were found, which provided a new idea for improving the penetration depth of jet. The influence of liner material, cone angle and stand-off on jet forming and penetration was also studied by orthogonal optimization experiment, and the structural parameters with the best penetration performance were obtained. The results show that the pressure at the convergence point increases first and then decreases during the formation of the jet of the three-layer liner. The pressure at the convergence point when the three-layer liner material is from low impedance to high impedance from the outside to the inside is much larger than the pressure at the convergence point from high impedance to low impedance. When the three-layer liner material is Al 2024-Copper–Tantalum from the outside to the inside, the pressure at the convergence point of the three-layer liner at different times is higher than that of the double-layer liner and the single-layer liner. Reasonable matching of different impact impedance materials in the three-layer liner can greatly improve the pressure value of the detonation wave acting on the cone liner. The maximum pressure at the convergence point on the axis is 39.10 GPa, which is 22.00% higher than that of the double-layer liner at the convergence point, and 53.03% higher than that of the single-layer liner at the convergence point. The orthogonal design test scheme is simulated and analyzed. The penetration depth is taken as the observation index, and the range analysis is adopted. The results show that the material matching of the three-layer liner has the greatest influence on the depth of the jet penetrating the target plate, followed by the cone angle of the three-layer liner. Relatively speaking, the stand-off has the least influence on the result. Reasonable matching of materials with different impact impedances in the three-layer liner can maximize the penetration depth of the jet into the target plate.

## Introduction

With the continuous improvement of various armor protection technologies, the armor protection performance has increased significantly, and then the damage performance requirements of armor-piercing ammunition have also increased. As the main damage component of anti-armor, the shaped charge warhead crushes the liner by the shaped charge effect, forming a high-speed metal jet to damage the target. The energy utilization rate of jet formed by single-layer liner is low, and the penetration effect is poor. The liners of different materials are added to the liners to form double-layer or multi-layer liners, which can enhance the ability of armor breaking^1–3^.

In order to improve the power of shaped charge, a lot of research has been carried out on charge, detonation waveform, liner material and liner structure. The three-layer liner has many advantages due to the use of new materials and new structures. For example, the head speed of the jet is large, and the tensile length of the jet is large. For the jet formation of the three-layer liner, only some preliminary experimental studies have been carried out, and there are few reports on the analysis model^1,4^.

Therefore, a new type of three-layer liner structure is designed in this paper, the 2014 version of AUTODYN software was used to study the jet forming process and penetration performance.The mechanism of shaped jet forming of three-layer liner is mainly studied, and the reason why the three-layer liner improves the penetration depth compared with the ordinary liner is explained. The influence of three-layer liner on the propagation of detonation wave and the change of pressure when detonation wave acts on the liner are found, which provides a new idea for improving the penetration depth of jet. The influence of liner material, cone angle and stand-off on jet forming and penetration was studied by orthogonal optimization experiment, and the structural parameters with the best penetration performance were obtained^5,6^. This is of great significance for improving the comprehensive damage power and combat effectiveness of the new shaped charge warhead compared with the traditional shaped charge.

## Numerical calculation model

### Establish numerical calculation model

**Figure 1:**
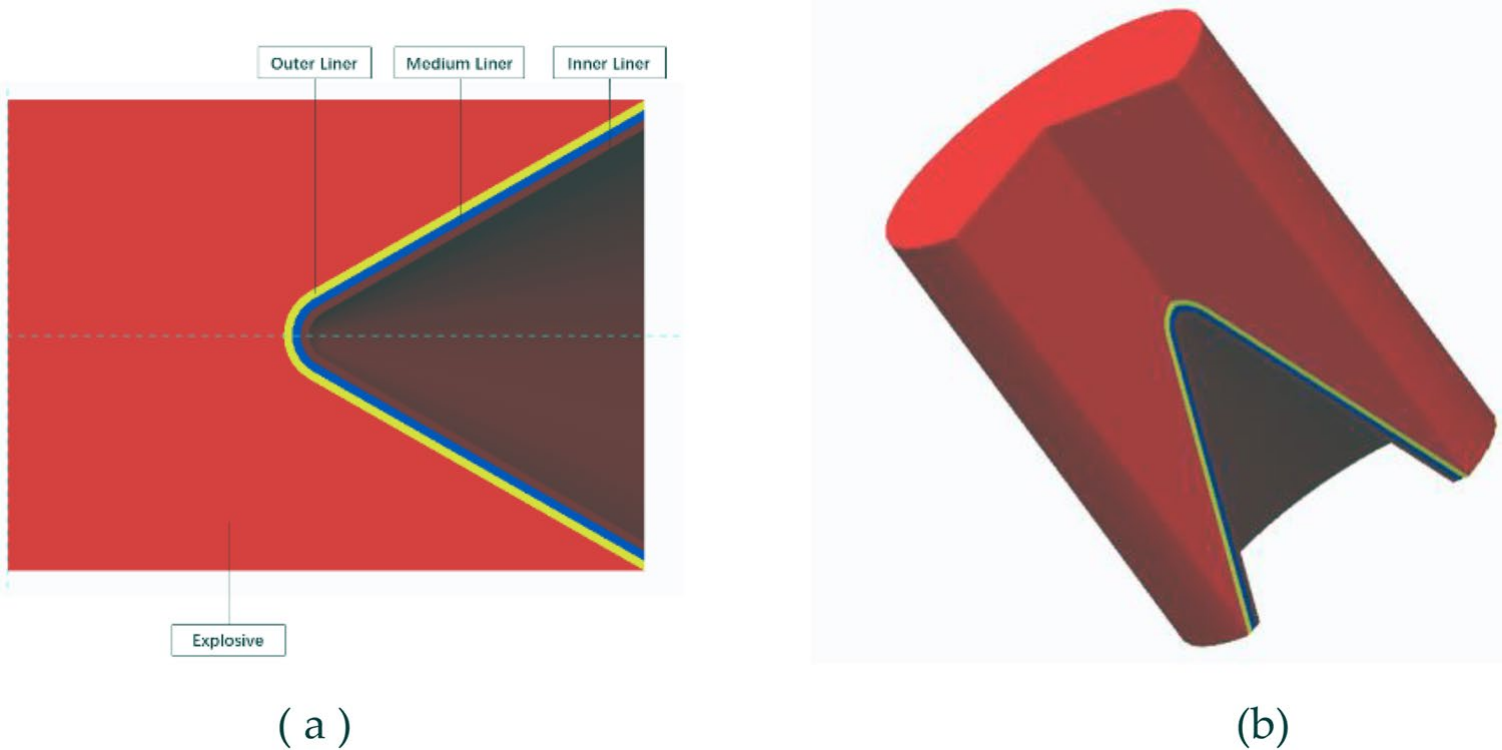
Shaped warhead model. (**a**) Structure diagram of shaped warhead. (**b**) Geometric model of shaped charge.

In order to better study the jet forming, this paper adopts the shellless shaped charge structure and the equal wall thickness conical cover, in which the cone top is rounded, as shown in Fig. 1.Among them, the wall thickness of the liner is $\delta$ = 3 mm, the charge diameter is ${D}_{k}$ = 56 mm, and the charge height is H = 75 mm.Taking the cone angle of the liner $2\alpha$ = 60° as an example, the structure of the shaped warhead is as follows.

**Figure 2:**
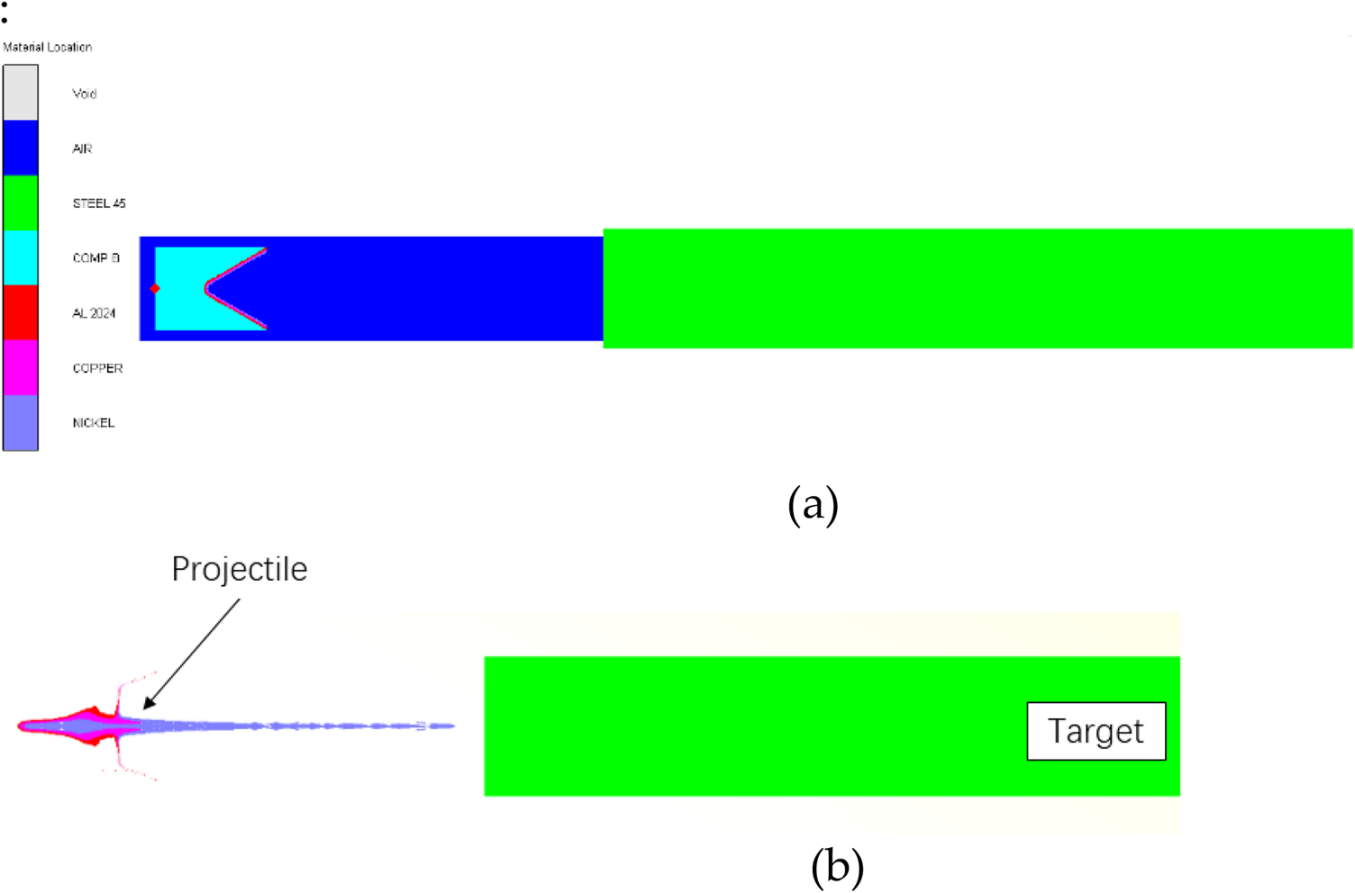
Numerical simulation model: (**a**) The initial conditions of shaped charge with three-layer liner. (**b**) Jet penetrating steel plate model.

In the numerical simulation, the influence of the shell is ignored, and the detonation mode is initiated by the center point. Due to the symmetry of the structure, the two-dimensional axisymmetric method is used to establish the model. The model adopts the mm-mg-ms unit system. The Euler algorithm is used for air, explosives and liners. The ' FLOW OUT ' boundary condition is added to the air boundary to eliminate the boundary effect^7^. The Euler mesh size is 0.25 × 0.25. The finite element model is shown in Fig. 2 :

### Material model

In Autodyn numerical simulation software, the change of material parameter state is usually reflected by state equation and strength model. The state equation describes the relationship between the volume, pressure and internal energy of the material while giving the initial response of the material. The strength model describes a nonlinear elastic–plastic response. In the case of dynamic loading, plastic deformation occurs if the load strength of the solid material with elastic initial response exceeds the yield stress of the material itself.

The state equation of air is Ideal Gas, which is derived from Boyle and Gay Lussac 's law. The state equation is applicable to most occasions involving gas motion, and its expression is^8,9^ :

1 $${\text{P}}_{{\text{A}}} = \left( {{\upgamma } - 1} \right){\uprho }_{{\text{A}}} {\text{E}}_{{\text{A}}}$$

In the equation, ${\uprho }_{{\text{A}}}$ is the air density, which is $1.225\times {10}^{-3}$ g/${{\text{cm}}}^{3}$; $\upgamma$ is the adiabatic index, which is 1.4; ${{\text{E}}}_{{\text{A}}}$ = 206.8 kJ/${{\text{m}}}^{3}$.

Comp B is selected as the explosive, and the ' Jones–Wilkins–Lee ' (JWL) state equation is used to describe the high-energy detonation and the expansion process of detonation gas during the ex $plosion$ process. The expression is :

2 $${{\text{P}}}_{{\text{E}}}={\text{A}}\left(1-\frac{\upomega }{{{\text{R}}}_{1}{\text{V}}}\right){{\text{e}}}^{-{{\text{R}}}_{1}{\text{V}}}+{\text{B}}\left(1-\frac{\upomega }{{{\text{R}}}_{2}{\text{V}}}\right){{\text{e}}}^{-{{\text{R}}}_{2}{\text{V}}}+\frac{\upomega {{\text{E}}}_{0}}{{\text{V}}}$$

In formula (2) : A,B, ${{\text{R}}}_{1}$, ${{\text{R}}}_{2}$, $\upomega$ are input parameters; ${{\text{P}}}_{{\text{E}}}$, ${{\text{E}}}_{0}$ and V are respectively the pressure of explosive products, the internal energy per unit volume and the relative volume (the volume of detonation products produced by the charge per unit volume). Specific parameters are shown in Table 1 below.

**Table 1:** Material parameters of Comp B.

$\uprho \, \left({\text{g}}/{{\text{cm}}}^{3}\right)$	E (kJ⋅m^−3^)	${\text{A}} \, \left({\text{Gpa}}\right)$	${\text{B}} \, \left({\text{Gpa}}\right)$	${{\text{R}}}_{1}$	${{\text{R}}}_{2}$	$\upomega$
1.717	$8.50\times {10}^{6}$	524.23	7.678	4.2	1.1	0.34

The material of the liner is AL 2024, Copper, Nickel and Tantalum. The equation of state is Shock, which is a kind of Mie-Gruneisen equation of state. Shock equation can determine the relationship between any two of the five variables of impact velocity U, particle velocity ${{\text{u}}}_{{\text{p}}}$, pressure P, material density $\uprho$ and energy e. For most solids and liquids, there are the following empirical formulas for impact velocity U and particle velocity ${{\text{u}}}_{{\text{p}}}$^10,11^ :

3 $${\text{U}}={{\text{c}}}_{0}+{{\text{su}}}_{{\text{p}}}$$

To establish the Shock equation of state :

4 $${\text{p}}={{\text{P}}}_{{\text{H}}}+\mathrm{\rho \gamma }({\text{e}}-{{\text{e}}}_{{\text{H}}})$$

5 $${P}_{H}=\frac{{\rho }_{0}{c}_{0}^{2}u(1+u)}{{\left[1-\left(s-1\right)u\right]}^{2}}$$

6 $${e}_{H}=\frac{1}{2}\frac{{P}_{H}}{{P}_{0}}(\frac{\mu }{1+\mu })$$

where $\mathrm{\gamma \rho }={\upgamma }_{0}{\uprho }_{0}$= constant, $\uprho$ is the material density, $\upgamma$ is the Gruneisen coefficient, usually taking the approximate value 2. ${{\text{P}}}_{{\text{H}}}$ denotes Hugoniot pressure and ${{\text{e}}}_{{\text{H}}}$ denotes Hugoniot energy. The Shock state equation gives the limit value of compression. At this time, the pressure approaches infinity, and the denominator in Formula ( 5 ) becomes zero, that is :

7 $$1-\left({\text{s}}-1\right)\upmu =0$$

When the jet penetrates the target plate, the target plate adopts STEEL 45, and its strength model and failure model adopt Johnson–Cook. The specific parameters are shown in Table 2 :

**Table 2:** Material parameters of STEEL 45.

Materials	$\uprho ({\text{g}}/{{\text{cm}}}^{3})$	G (Gpa)	A (Gpa)	B (Gpa)	N	c	m	${{\text{T}}}_{{\text{m}}}({\text{K}})$
STEEL 45	7.85	76.0	0.507	0.32	0.28	0.064	1.06	1793

## Analysis of detonation wave propagation process of three-layer liner

The detonation wave is a strong shock wave propagating along the explosive. After it passes through, the explosive is immediately excited by a high-speed chemical reaction due to a strong impact, forming a high-temperature and high-pressure detonation product and releasing a large amount of chemical reaction heat energy. These energy is used to support the detonation wave to impact and compress the next layer of explosives. Therefore, the detonation wave can propagate without attenuation. After the initiation of the explosive, the detonation wave propagates forward in the form of spherical wave, which rapidly overwhelms the liner and closes it on the axis to form a metal jet with higher density and energy. As a structural composite liner, the structure of the three-layer liner has an effect on the propagation direction of the detonation wave, the waveform of the detonation wave and the pressure value acting on the liner^12^.

### Influence of impact impedance of three-layer liner material on detonation wave propagation

When the detonation wave passes through the liner element, the impact pressure of the detonation wave drives the liner element. At the same time, the detonation wave will also transmit and reflect between the liners. The transmitted detonation wave will drive the next layer of the liner, and the reflected detonation wave will continue to act on the previous layer of the liner, so the middle layer of the liner will be driven by complex stress waves. The selection of liner materials with different impact impedances will directly affect the formation of the three-layer liner jet^13^. The impact impedance refers to the product of the medium density and the shock wave velocity. It is the dynamic stiffness of the medium. Its physical meaning is the pressure required for the medium to obtain a velocity of 1m/s under the impact load.

According to the existing stress wave theory, the propagation of stress wave between the micro-elements of the three-layer liner follows the formula of stress wave propagation at the interface of different media, that is:

8 $${{\text{P}}}_{{\text{T}}}=\frac{2}{1+\mathrm{\alpha }}{{\text{P}}}_{1}$$

9 $${{\text{P}}}_{{\text{R}}}=\frac{1-\mathrm{\alpha }}{1+\mathrm{\alpha }}{{\text{P}}}_{1}$$

**Figure 3:**
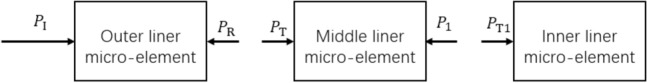
The force diagram of the three-layer liner micro-element.

In the formula: ${{\text{P}}}_{1}$ is the incident pressure ; ${{\text{P}}}_{{\text{T}}}$ is the transmission pressure ; ${{\text{P}}}_{{\text{R}}}$ is the reflection pressure ; $\mathrm{\alpha }$ is the impact impedance ratio of the front and rear materials. The propagation of stress waves between the three-layer liner micro-elements is shown in Fig. 3.

After the initiation of the warhead, the detonation wave reaches the liner and crushes the bottom of the liner. In the crushing stage of the three-layer liner, the detonation product after the explosion is driven by high temperature and high pressure. The surface normal of the explosive is scattered outward, and the effective charge near the liner will transfer its own explosion energy to the three-layer micro-unit. This transfer is completed in the form of a detonation wave sweeping through the liner. After a period of time, the infinitesimal obtains the limit collapse velocity and collapse angle, and then converges to the axis. This convergence motion is also called collapse motion^14–16^.

The propagation of detonation wave and its effect on the liner can be approximately expressed by the pressure value and the shape of the isobaric line. In order to study the propagation mode of detonation wave in the shaped charge of three-layer liner and compare the propagation law of detonation wave by impact impedance matching of three-layer liner, this section selects the three-layer liner material from the outside to the inside as low impedance to high impedance, high impedance to low impedance for analysis and research. The impact impedance parameters of the liner material are as follows Table 3^15^ :

**Table 3:** Basic parameters of different materials.

Materials	Density (${\text{g}}/{{\text{cm}}}^{3}$)	Sound velocity(m/s)	Impedance of impact(${\text{N}}\cdot {\text{s}}/{{\text{m}}}^{3}$)
AL 2024	2.785	5328	$1.48\times {10}^{7}$
COPPER	8.93	3940	$3.52\times {10}^{7}$
NICKEL	8.874	4602	$4.08\times {10}^{7}$
TANTALUM	16.654	3414	$5.69\times {10}^{7}$

The comparison scheme of three-layer shaped charge is shown in Table 4.

**Table 4:** Three kinds of shaped charge parameters.

From low impedance to high impedance						
A	56	75	Comp B	AL2024/COPPER/NICKEL	1/1/1	60
B	56	75	Comp B	AL2024/COPPER/TANTALUM	1/1/1	60
C	56	75	Comp B	AL2024/ NICKEL/TANTALUM	1/1/1	60
D	56	75	Comp B	COPPER/NICKEL/TANTALUM	1/1/1	60
From high impedance to low impedance						
e	56	75	Comp B	NICKEL/COPPER/AL2024	1/1/1	60
f	56	75	Comp B	TANTALUM/COPPER/AL2024	1/1/1	60

**Figure 4:**
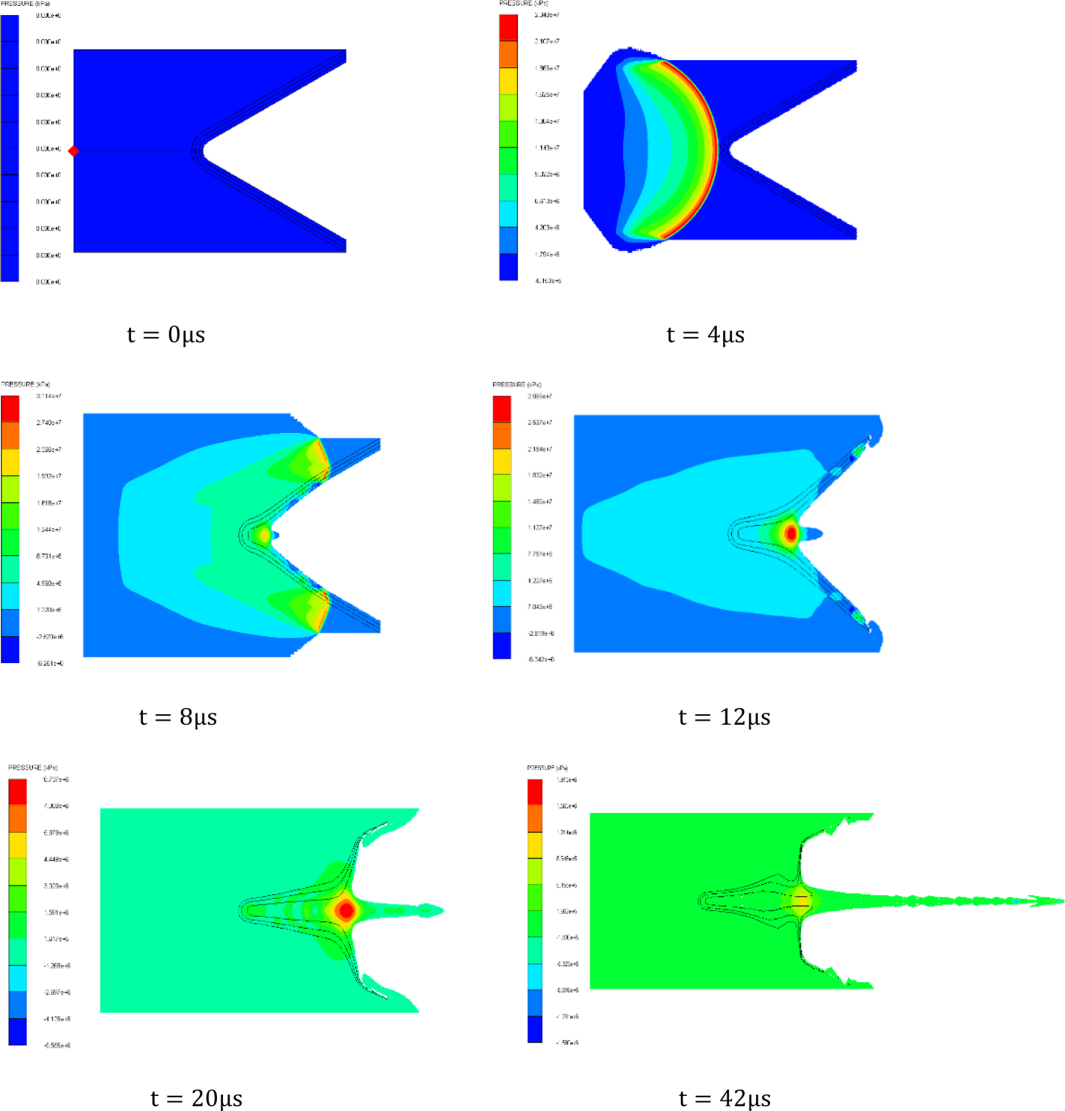
Propagation process of detonation wave in three-layer liner.

**Figure 5:**
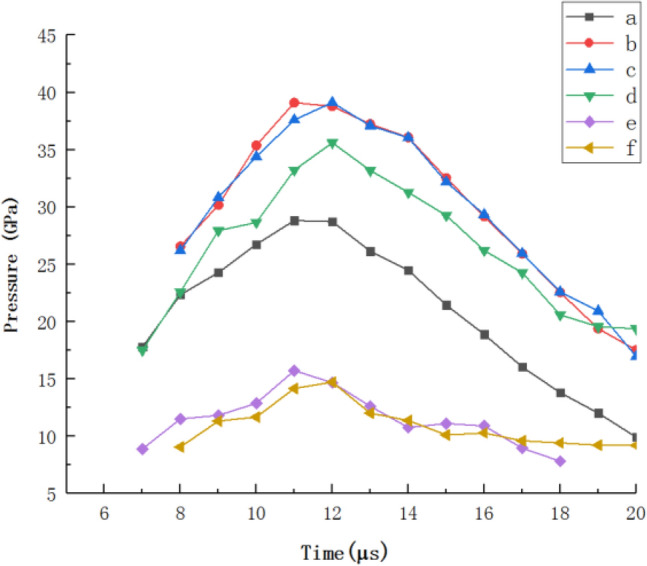
The pressure at the convergence point at different moments.

The following figure takes the three-layer liner material from the outside to the inside as AL 2024-COPPER–Nickel as an example to show the propagation process of detonation wave in shaped charge, the propagation process of detonation wave in three kinds of shaped charge and the pressure at the convergence point of the liner under different impedance matching as shown in Figs. 4 and 5.

It can be seen from Fig. 4 that after the detonation of the explosive, the detonation wave propagates forward along the axis in the form of spherical wave. When t = 4 $\mathrm{\mu s}$, the detonation wave reaches the top of the cone liner and begins to act on the three-layer liner, and then the outer liner is squeezed to the axis under the action of the detonation product. When t = 8 $\mathrm{\mu s}$, the inner liner is rapidly squeezed under the action of the detonation wave, converging at the axis, and initially forming the jet head. The outer liner and the middle liner are further squeezed and deformed. When t = 42 $\mathrm{\mu s}$, the inner liner is further stretched to form a jet, a part of the middle liner forms a jet, a part of the middle liner forms a pestle, and the outer liner all forms a pestle.

It can be seen from Fig. 5 that the pressure at the convergence point increases first and then decreases during the formation of the jet of the three-layer liner. The pressure at the convergence point when the three-layer liner material is from low impedance to high impedance from the outside to the inside is much larger than the pressure at the convergence point from high impedance to low impedance. This is because the impact impedance of the liner is gradually increasing from the outer layer to the inner layer, so the transmission pressure will be amplified step by step. When the three-layer liner material is Al 2024-Copper–Tantalum from the outside to the inside, the pressure at the convergence point is the largest, reaching a peak of 39.10 Gpa at t = 11 μs.

### Comparative analysis

**Figure 6:**
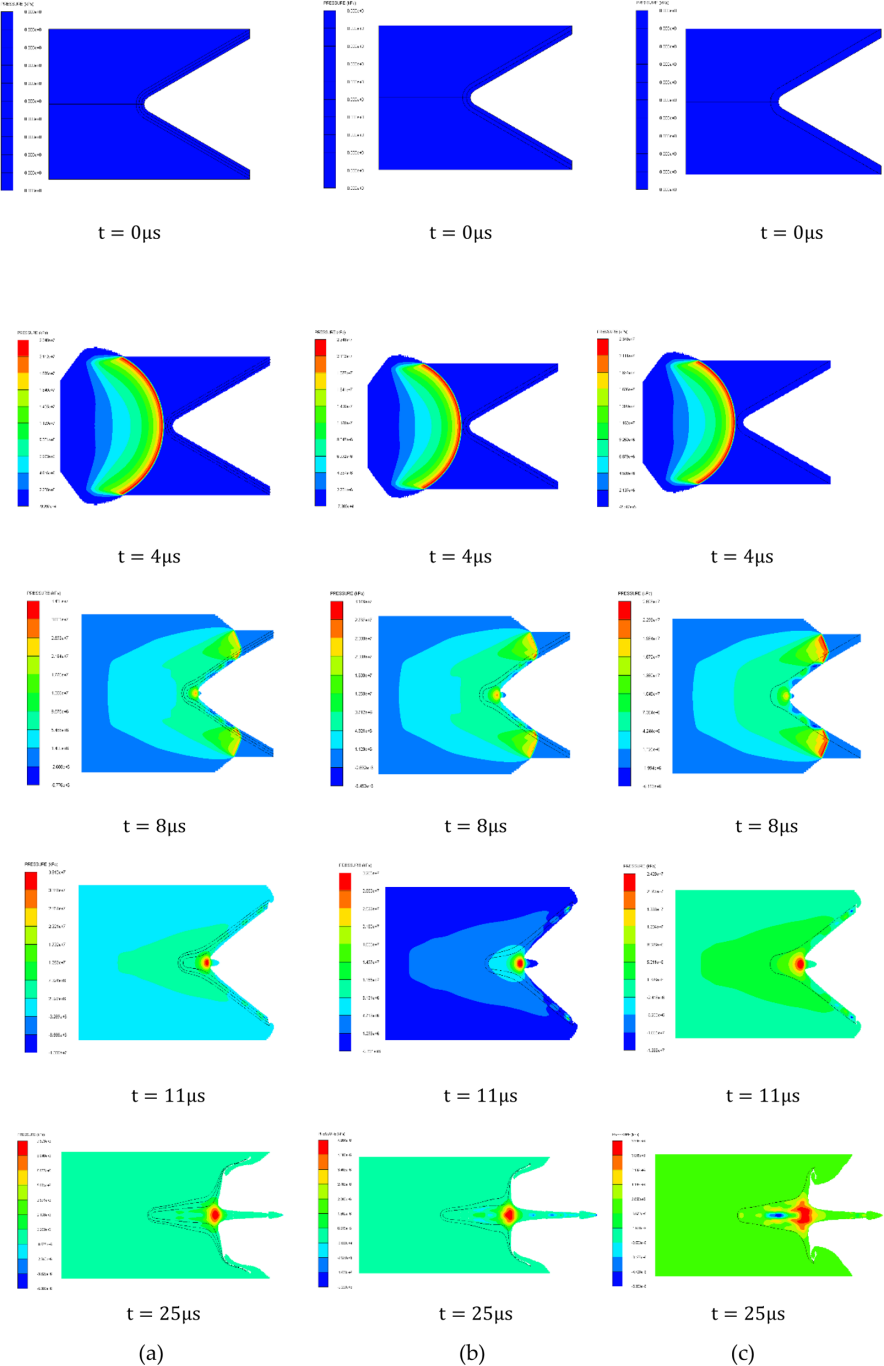
Detonation wave propagation process. (**a**) Three-layer liner. (**b**) Double-layer liner. (**c**) Single-layer liner.

**Figure 7:**
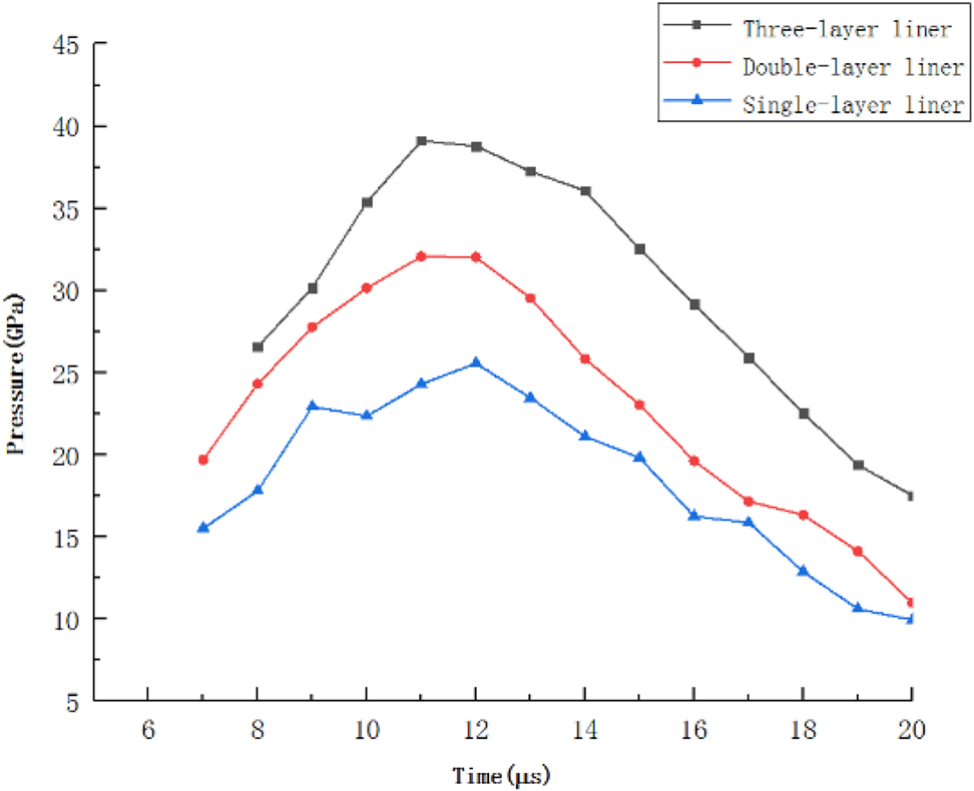
The pressure at the convergence point at different times.

The propagation of detonation wave and its effect on the liner can be approximately expressed by pressure value and isobaric line shape. In order to study the propagation mode of detonation wave in three-layer liner shaped charge, the propagation law of detonation wave in traditional single-layer liner shaped charge and double-layer liner shaped charge is compared. The parameters of the three shaped charge are shown in Table 5 below^17–19^. The propagation process of detonation wave in three kinds of shaped charge and the pressure at the convergence point are shown in Figs. 6 and 7.

**Table 5:** Three kinds of shaped charge parameters.

Scheme	Charge diameter/(mm)	Charge length/(mm)	Explosive	Materials	Wall thickness	cone angle (°)
Three-layer liner	56	75	Comp B	AL2024/COPPER/TANTALUM	1/1/1	60
Double-layer Liner	56	75	Comp B	AL2024/COPPER	1.5/1.5	60
single-layer liner	56	75	Comp B	COPPER	3	60

It can be seen from Fig. 6 above that the propagation of detonation wave in the multi-layer liner is similar to that of the single-layer liner. After the detonation of the explosive, the detonation wave reaches the bottom of the cone liner and crushes the liner to form a jet. When t = 8 μs, the inner liner is rapidly squeezed under the action of detonation wave, converging at the axis, and the jet head is initially formed. At this time, the maximum pressure on the inner side of the liner is greater than the pressure at the convergence point. At this time, the maximum pressure on the inner side of the three-layer liner is 34.10 GPa, the pressure on the inner side of the double-layer liner is 31.46 GPa, and the pressure on the inner side of the single-layer liner is 26.08 GPa. The inner pressure of the side wall of the three-layer liner is 8.39% higher than that of the double-layer liner at t = 8 μs, and 30.75% higher than that of the single-layer liner.

It can be seen from Fig. 7 that the pressure at the convergence point of the three-layer liner at different times is higher than that of the double-layer liner and the single-layer liner. Reasonable matching of the materials with different impact impedances in the three-layer liner can greatly improve the pressure value of the detonation wave acting on the cone liner. As shown in the above diagram, when t = 11 μs, the maximum pressure at the convergence point on the axis is 39.10 GPa, which is 22.00% higher than the maximum pressure at the convergence point of the double-layer liner at t = 11 μs, and 53.03% higher than the maximum pressure at the convergence point of the single-layer liner at t = 12 μs. As the cone cover continues to collapse, the pressure at the convergence point gradually decreases. It is worth noting that in the process of pressure drop at the convergence point, the pressure at the convergence point of the three-layer liner is always greater than the pressure at the convergence point of the double-layer liner and the single-layer liner. It can be seen that the addition of the three-layer liner has an effect on the propagation of the detonation wave and can increase the pressure value of the detonation wave acting on the cone.

## Jet penetration performance analysis

### Orthogonal design

The main target of the fragment warhead is the armor protection target, which not only requires it to have sufficient armor-piercing ability, but also requires the jet to have a corresponding aftereffect after penetrating the armor. Therefore, it is particularly important to design the warhead power. As a new type of charge structure, the penetration power of the three-layer shaped charge is affected by many factors, including the impedance matching of the three-layer shaped charge material, the cone angle of the shaped charge and the stand-off.

Orthogonal experimental method is a kind of scientific arrangement and analysis of multi-factor test method, which can solve the problem of multi-factor, multi-level and multi-index. It selects the representative level from the combination of each factor test level and uses the appropriate orthogonal table to carry out the combination test. Through the processing and analysis of the test results, the influence degree of each factor on the evaluation results can be obtained, and the optimal factor level combination can be found accordingly, so as to point out the further test direction. Its detailed introduction and orthogonal table design can participate in the literature^20–22^.

Based on the analysis of the influence of the above factors on the results of jet penetration performance, in order to further obtain the influence degree of each factor on the evaluation results, the optimal combination of factor levels is also found, which includes three factors : the impact impedance matching of the material of the three-layer liner, the cone angle and the stand-off of the liner. Four levels of each factor were selected to conduct orthogonal design experiments on the formation of three-layer liners. The penetration depth of the jet was used as the evaluation index to study the results. The best three-layer liner structure was obtained by synthesizing various influencing factors. After the preliminary optimization calculation, the values of the above factors are gradually reduced, and the level values of each factor are shown in Table 6^17,22^.

**Table 6:** The level table of each factor optimized by orthogonal design.

LEVEL	Factor		
	Materials (m)	Standoff (h)	Cone angle ($\mathrm{\alpha })$/°
1	AL2024/COPPER/NICKEL	2D	50
2	AL2024/COPPER/TANTALUM	3D	55
3	COPPER/NICKEY/TANTALUM	4D	60
4	NICKEL/COPPER/AL2024	5D	65

According to the three factors of material matching, stand-off and cone angle of three-layer liner, four levels are set for each factor, and L(16) ${4}^{3}$ orthogonal table is selected to design the orthogonal test scheme. The most factors that can be arranged in this orthogonal table are 3, which meets the conditions of this design. According to the factors and levels listed in Table 6, the orthogonal table is shown in Table 7 below.

**Table 7:** Orthogonal test scheme and numerical simulation results.

Scheme	Materials (m)	Standoff (h)	Cone angle ($\mathrm{\alpha })$/°	Penetration depth(mm)
1	AL2024/COPPER/NICKEL	2D	50	446.0
2	AL2024/COPPER/NICKEL	3D	55	403.8
3	AL2024/COPPER/NICKEL	4D	60	477.5
4	AL2024/COPPER/NICKEL	5D	65	365.3
5	AL2024/COPPER/TANTALUM	2D	55	404.0
6	AL2024/COPPER/TANTALUM	3D	50	366.8
7	AL2024/COPPER/TANTALUM	4D	65	446.5
8	AL2024/COPPER/TANTALUM	5D	60	501.8
9	COPPER/NICKEY/TANTALUM	2D	60	370.5
10	COPPER/NICKEY/TANTALUM	3D	65	439.3
11	COPPER/NICKEY/TANTALUM	4D	50	340.0
12	COPPER/NICKEY/TANTALUM	5D	55	345.3
13	NICKEL/COPPER/AL2024	2D	65	376.8
14	NICKEL/COPPER/AL2024	3D	60	396.5
15	NICKEL/COPPER/AL2024	4D	55	315.8
16	NICKEL/COPPER/AL2024	5D	50	313.0

### Results analysis and optimization scheme determination

In order to distinguish the primary and secondary relations of various factors and their interactions, it is necessary to analyze the test results. Usually, the range analysis method is used to obtain the primary and secondary order of the influence of various factors on the index, so as to determine the optimal structure.

The range method is used to analyze the data of each group. The optimal level of this factor and the combination of the levels of each factor are judged by the average number of the corresponding indexes of the n level of the j column factor in the orthogonal design. The formula is^5,18,23^ :

9 $${{\text{R}}}_{{\text{j}}}={\text{max}}\left({{\text{k}}}_{{\text{j}}1},\cdots ,{{\text{k}}}_{{\text{jn}}}\right)-{\text{min}}({{\text{k}}}_{{\text{j}}1},\cdots ,{{\text{k}}}_{{\text{jn}}})$$

In the formula : ${{\text{k}}}_{{\text{j}}}$ is the average of the sum of the indexes corresponding to the n level of the j column factors ; ${{\text{R}}}_{{\text{j}}}$ represents the range of j column factors under each level index, which reflects the change range of the test index when the level of the j column factor changes. The larger the ${{\text{R}}}_{{\text{j}}}$, the greater the influence of the factor on the test index, and the higher the master–slave order.

**Figure 8:**
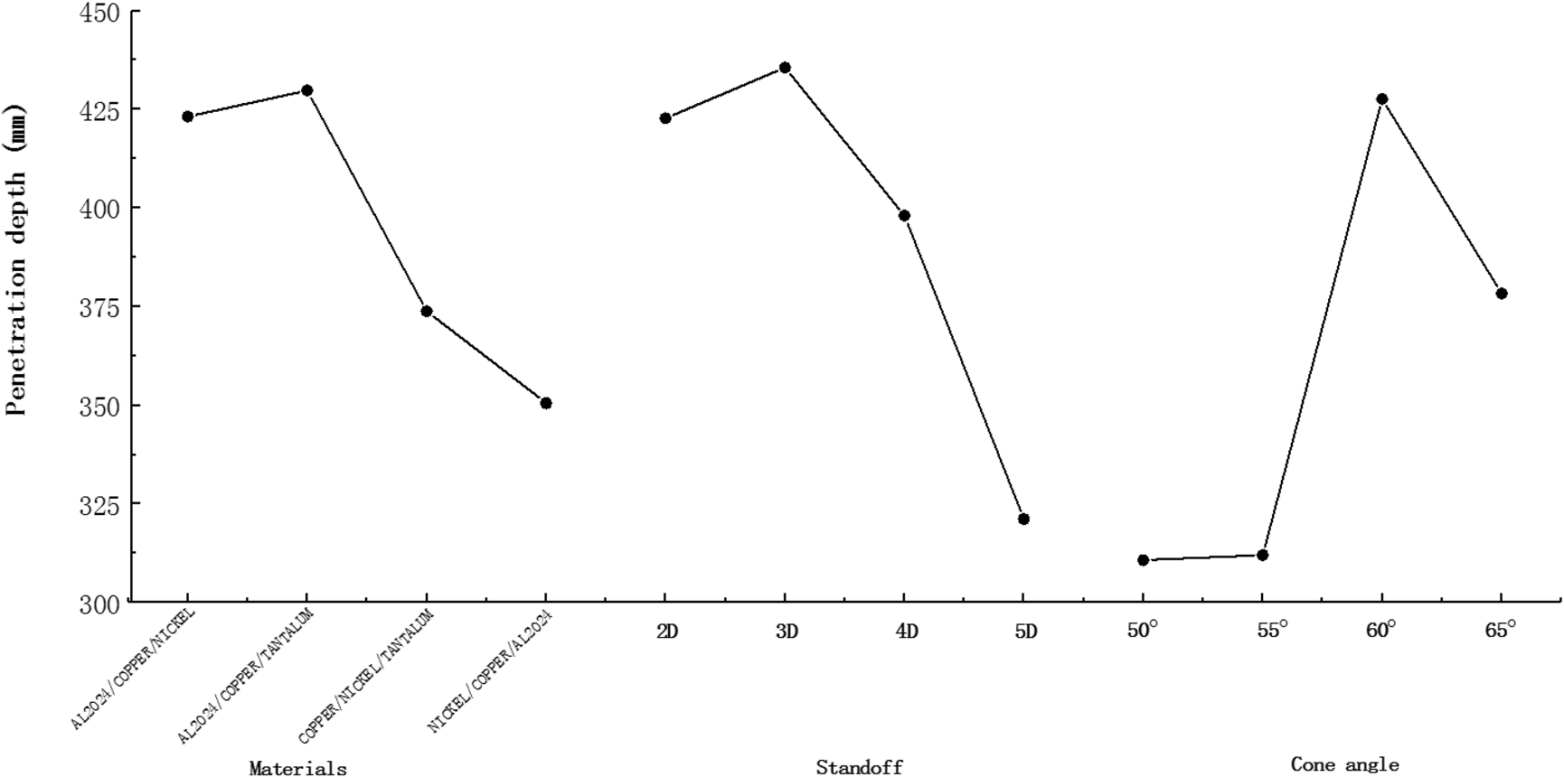
The trend chart of the influence of each factor level on the penetration depth.

According to the orthogonal numerical test results of Table 7, for each evaluation index, the statistical average is carried out according to three factors and four levels, and then the range analysis is carried out on the statistical average of each factor and four levels, and the analysis results shown in Table 8 are obtained. The results are rounded to the maximum and retained to two decimal places. In the table, K is the statistical average, and R is the range of the statistical average, which reflects the influence of the level change of the factors selected in the column on the index. According to the range analysis, the influence trend of each factor on the penetration depth is drawn as shown in Fig. 8^24,25^.

**Table 8:** Statistical analysis of numerical simulation results.

Level	Materials (m)	Standoff (h)	Cone angle ($\mathrm{\alpha })$/°
K			
1	423.15	399.32	366.45
2	429.77	401.60	367.22
3	373.77	394.95	436.57
4	350.52	381.35	406.97
Best level	AL2024/COPPER/TANTALUM	3D	60〫
R	79.25	20.25	70.13

It can be seen from Table 8 that the range of the three factors for the evaluation index of the three-layer liner material matching m, the stand-off h, and the three-layer liner cone angle α is 79.25, 20.25 and 70.13, respectively. From the range size, it can be seen that for the penetration depth of the jet, the primary and secondary factors are $m>\alpha >h$. It can be judged that the matching of the three-layer liner material has the greatest influence on the penetration depth of the jet into the target plate, followed by the cone angle of the three-layer liner. Under the comparison of the penetration depth of the three factors on the three-layer liner, the stand-off has the least influence on the result. From the range size, it can be seen that the influence of the material matching of the three-layer liner on the penetration depth of the jet is 3.9 times that of the stand-off on the penetration depth of the jet. The results show that the reasonable matching of the materials with different impact impedances in the three-layer liner can maximize the penetration depth of the jet to the target plate. At the same time, according to the Fig. 8 above, for the three setting factors, the penetration ability of the jet increases first and then decreases with the change of the single factor level, which can predict the optimal result of the change combination of the influencing factors on the penetration depth of the jet, which can provide reference for the early parameter screening of the optimal design of the charge structure. For the liner structure and charge structure in this study, the combination of ${m}_{2}{h}_{2}{\alpha }_{3}$ can obtain the maximum penetration depth.

**Figure 9:**
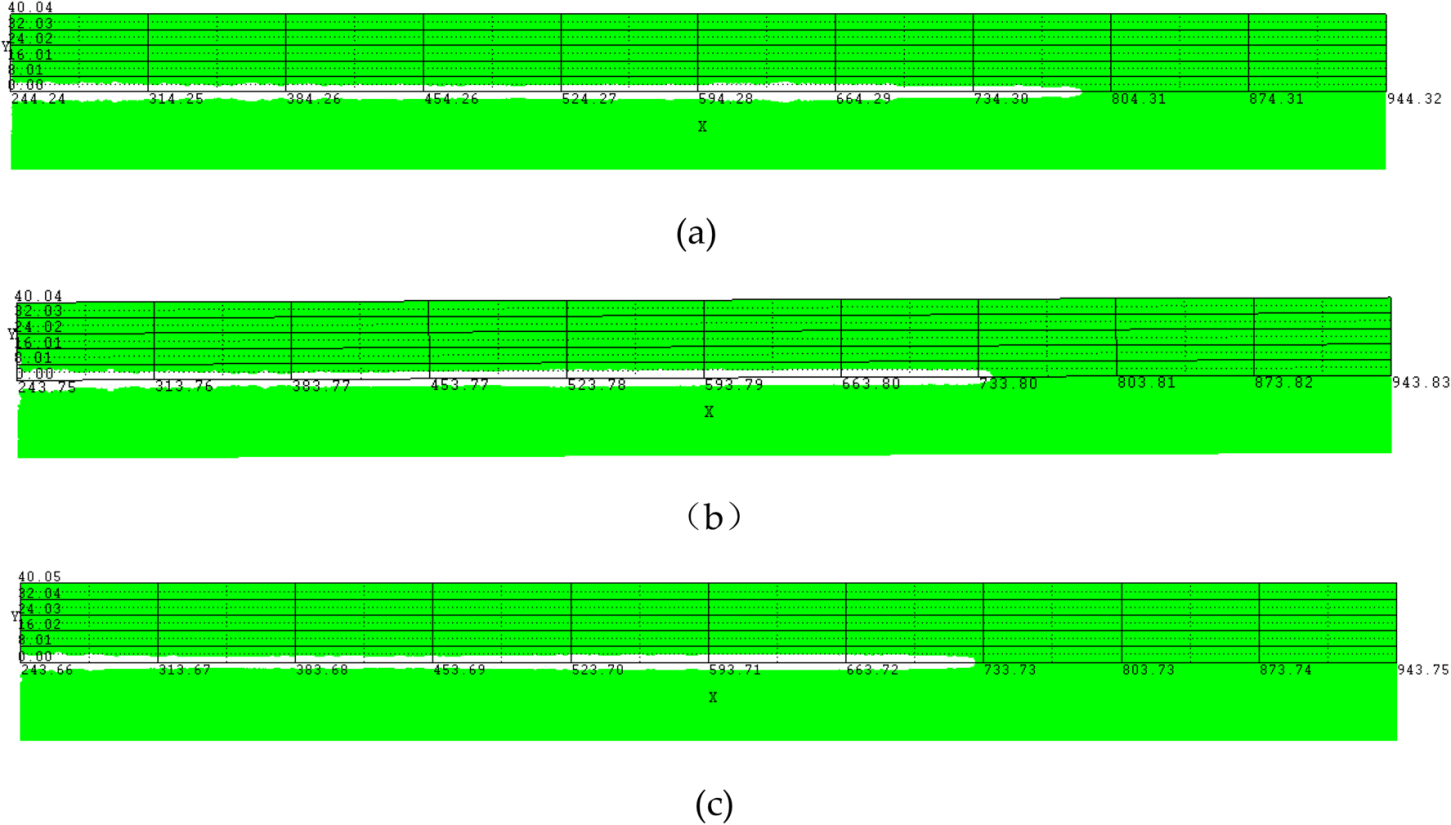
Simulation of jet penetration depth (**a**) Three-layer liner. (**b**) Double-layer liner. (**c**) Single layer liner.

The damage simulation test of the target plate is carried out on the optimal three-layer liner structure obtained above. The specific parameters of the three-layer liner are as follows: the wall thickness of the liner is $\updelta$ = 3 mm, the cone angle of the liner is 2 $\mathrm{\alpha }$ = 60°, the charge diameter is ${{\text{D}}}_{{\text{K}}}$ = 56 mm, the charge height is H = 75 mm, the target plate material is steel 45, the specific size is 700 mm in length and 80 mm in diameter. Compared with the double-layer liner and the single-layer liner with the same structure, the maximum penetration depth of different types of liners on the target plate is as follows: Table 9 and Fig. 9 show :

**Table 9:** Jet penetration depth of different liner types.

Type	Materials	Wall thickness	Cone angle (°)	Penetration depth(mm)
Three-layer liner	AL2024/COPPER/TANTALUM	1/1/1	60	547.4
Double-layer liner	AL2024/COPPER	1.5/1.5	60	518.0
Single-layer liner	COPPER	3	60	486.5

From the above Table 9, it can be seen that the maximum penetration depth of the damage of the three-layer liner to the target plate obtained by the best scheme is 547.4 mm. Compared with the double-layer liner, the penetration depth of the jet is increased by 5.68%. Compared with the single-layer liner, the penetration depth of the jet is increased by 12.52%.

## Result

In this paper, a new three-layer liner structure is designed, and its jet forming process and penetration performance are studied. The mechanism of shaped jet forming of three-layer liner is mainly studied, and the reasons for improving the penetration ability of three-layer liner are explained. The influence of three-layer liner on the propagation of detonation wave and the change of pressure when detonation wave acts on the liner are found, which provides a new idea for improving the penetration depth of jet. The influence of liner material, cone angle and stand-off on jet forming and penetration was also studied by orthogonal optimization experiment, and the structural parameters with the best penetration performance were obtained. This is of great significance for improving the comprehensive damage power and combat effectiveness of the new shaped charge warhead compared with the traditional shaped charge. The main conclusions are as follows:

1. The pressure at the convergence point increases first and then decreases during the formation of the jet by the three-layer liner. The pressure at the convergence point when the three-layer liner material is from low impedance to high impedance from the outside to the inside is much larger than the pressure at the convergence point from high impedance to low impedance. This is because the impact impedance of the liner is gradually increasing from the outer layer to the inner layer, so the transmission pressure will be amplified step by step.
2. When the material of the three-layer liner is Al 2024-Copper–Tantalum from the outside to the inside, the pressure of the three-layer liner at the convergence point at different times is higher than that of the double-layer liner and the single-layer liner. Reasonable matching of different impact impedance materials in the three-layer liner can greatly improve the pressure value of the detonation wave acting on the cone liner. The maximum pressure at the convergence point on the axis is 39.10 GPa, which is 22.00% higher than that of the double-layer liner at the convergence point, and 53.03% higher than that of the single-layer liner at the convergence point.
3. As the cone cover continues to collapse, the pressure at the convergence point gradually decreases. It is worth noting that in the process of pressure drop at the convergence point, the pressure at the convergence point of the three-layer liner is always greater than the pressure at the convergence point of the double-layer liner and the single-layer liner. It can be seen that the addition of the three-layer liner has an effect on the propagation of the detonation wave and can increase the pressure value of the detonation wave acting on the cone.
4. The orthogonal design test scheme is simulated and analyzed. The penetration depth is taken as the observation index, and the range analysis is used. The results show that the material matching of the three-layer liner has the greatest influence on the depth of the jet penetrating the target plate, followed by the cone angle of the three-layer liner. Relatively speaking, the stand-off has the least effect on the results. Reasonable matching of materials with different impact impedances in the three-layer liner can maximize the penetration depth of the jet into the target plate.

## Data Availability

The data used to support the findings of this study are included within the article.
